# Artificial Intelligence Technology for Food Nutrition

**DOI:** 10.3390/nu15214562

**Published:** 2023-10-27

**Authors:** Jinlin Zhu, Gang Wang

**Affiliations:** 1 State Key Laboratory of Food Science and Resources, Jiangnan University, Wuxi 214122, China; wanggang@jiangnan.edu.cn; 2 School of Food Science and Technology, Jiangnan University, Wuxi 214122, China

Food nutrition is generally defined as the heat energy and nutrients obtained from food by the human body, such as protein, fat, carbohydrates and so on. Throughout human life, nutrition has been the material basis for maintaining life activities, promoting growth and development, preventing chronic diseases, improving mental health and maintaining a good physiological state [1]. The 2018 Global Nutrition Report pointed out that a nutritional imbalance caused by an extreme lack or excess of nutrition is a worldwide nutritional security problem [2]. At present, there is sufficient research evidence to suggest that nutritional imbalance is the main risk factor leading to cardiovascular disease, diabetes and colorectal cancer [3,4]. Therefore, the principle of dietary nutrition balance pays attention to the diversity and rationality of food collocation, which has become an effective way for modern people to pursue health and quality life.

Artificial intelligence is used in science and engineering to expand upon human intelligence by building intelligent machines and intelligent computer programs. Technologies such as machine learning, neural networks and natural language processing contained within this field are leading the changes in the automobile, medical care, finance and other industries, and bringing about new opportunities [5,6,7,8]. In the late 2010s, artificial intelligence technology began to be extended into the field of food science and nutrition research and found new applications therein [9]. At present, with the new round of scientific and technological revolution and the in-depth evolution of industrial transformation, a new generation of cutting-edge technologies and equipment based on artificial intelligence is constantly infiltrating and integrating into the field of food nutrition, and driving the global food nutrition industry to develop rapidly in the direction of personalization, precision and intelligence [10]. Therefore, this paper summarizes the application and future development of artificial intelligence technology in the field of food nutrition in order to provide a reference for related research and applications.

At present, the incidence of obesity, cardiovascular diseases, diabetes and other chronic diseases in the population is rising. The effective prevention and treatment of diseases is a topic of widespread concern. Wang and colleagues [11] revealed the importance of a healthy diet in disease prevention through genome sequencing and machine learning. In addition, Wang and Min et al. [12] summarized the methods of automatic dietary nutrition assessment based on vision, and showed that dietary nutrition assessment based on artificial intelligence technology can provide scientific and reasonable intervention as well as guidance on how individuals can have healthy diets. Among them, dietary nutrition assessment based on artificial intelligence technology is divided into two frameworks: multi-stage and end-to-end. Within the framework of multi-stage dietary assessment, identifying and accurately measuring the composition of food is the premise of the method. Jaswanthi and colleagues [13] proposed a hybrid network based on GAN and CNN, which can predict the calories of food on the plate through three steps, namely, segmentation, recognition and recalculation. In contrast, the end-to-end dietary evaluation framework replaces these independent stages with a single neural network, which reduces error propagation and the joint optimization path [14]. Lu and colleagues [15] focused on the joint prediction of a depth map, semantic segmentation map and 3D food model from RGB food images via a single semi-supervised network without using depth information, and realized single-view meal evaluation for the first time.

Nutritional value refers to the degree to which the energy and nutrients contained in food can meet the nutritional needs of the human body. The nutritional value is not only related to the nutrient content in food, but is also closely related to the maturity of food, cooking technology and other factors. Artificial intelligence technology has powerful data processing and analysis capabilities, and it can mine valuable information by establishing the correlation of the data. Therefore, artificial intelligence technology has great potential in predicting and improving the nutritional value of food. Sandhu and colleagues [16] used a genetic algorithm and particle swarm optimization to predict the nonlinear functional correlation between cooking parameters and the nutritional quality index. By optimizing the combination of cooking parameters, the ratio of polyunsaturated fatty acids (PUFAs) to saturated fatty acids (SFAs) increased to 63.05%, thus enhancing the nutritional value of fried fish.

In recent years, people’s demand for nutritional safety and a high quality of food has been increasing every day. Food spoilage is the result of microbial growth and enzyme activity decomposition. Eating bad food by mistake will cause intestinal diseases such as gastroenteritis and pathological gastric ulcer, and even pose the risk of causing chronic poisoning. Ellis and colleagues [17] used a genetic algorithm and genetic programming to analyze the Fourier-transform infrared spectrum of beef, and used PLS linear regression and a machine learning method based on evolutionary computation to realize the rapid quantitative detection of rotten beef. This is a successful example of the exploration of artificial intelligence technology in food quality inspection.

Similarly, food adulteration is also a problem that cannot be ignored in the field of food nutrition. Food adulteration will not only affect the taste of food, but also reduce the quality and nutritional value of it. Hu and colleagues [18] combined Raman spectroscopy with support vector machines and other algorithms to detect low-concentration adulterated honey in a nondestructive and efficient way.

The continuous iterative development of new sensors and smart wearable devices further promotes the combination of artificial intelligence technology and food nutrition. Food recommendation based on artificial intelligence technology can provide more reasonable personalized dietary suggestions for people and help them to develop good eating habits and lifestyles [19]. However, food recommendation based on complex information such as taste preferences, perceptual differences, cognitive constraints and physical states is a challenging task. Yang and colleagues [20] learned the food preferences of users through a user interface based on a visual test and the proposed online learning framework, and made dietary recommendations for them. However, food recommendation should not only satisfy users’ food preferences, but also consider users’ health statuses. Therefore, Wang and colleagues [21] proposed a personalized health-conscious food recommendation scheme consisting of three parts: recipe retrieval, user health profile analysis and health-conscious food recommendation.

Interestingly, with changes in the consumption concept and lifestyles, people are paying more attention to the “emotional” value of food while pursuing the nutritional value of food, that is, the novelty and excitability of food flavor and taste [22]. Artificial intelligence technology can guide the macro or micro structure in the design of food, realize the innovation of food flavor and texture and achieve the unity of food sensory quality and health attributes. Fan and colleagues [23] used a machine learning algorithm to correlate volatile compounds with the sweetness and consumer preferences of strawberries, and predicted the key factors that could improve the sweetness and flavor of them, such as γ-dodecalactone and 6-methyl-5-hepten-2-one. It can be seen that it is of great significance for innovative food design and processing to screen potential key flavor compounds that indirectly regulate food flavor based on artificial intelligence technology.

In the future, with continuous progress in technology and the continuous expansion of application scenarios, the application of artificial intelligence in the field of food nutrition will be more extensive and in-depth. Incorporating more contextual information and building a more perfect data system are two methods that are expected to achieve more personalized nutrition recommendation and health management services. Moreover, the cross-integration of multiple disciplines and multiple fields will also make future food manufacturing more diverse, distinctive and connotative. Of course, processing and analyzing time-varying data or sequence data using perceptual interaction, mobile internet and other technologies may also be helpful for the sustainable and accurate design of food.

On the other hand, the rapid development process is bound to be accompanied by a series of complex challenges. Insufficient, inconsistent and inaccurate data will weaken the reliability of an algorithm. The interpretability and privacy security of artificial intelligence technology itself still need to be solved urgently. However, it is worth mentioning that the pressure and changes in the macro environment have also brought new opportunities for food technology, and more advanced food technologies are expected to make large contributions in global sustainable development, upgrading the consumer experience and optimizing enterprise cost efficiency.

To summarize, with the infiltration and integration between artificial intelligence technology and the food nutrition field, artificial intelligence not only provides more possibilities for food nutrition analysis and food innovation, but also provides key support for the reform of traditional food manufacturing.
